# Characteristics and prevalence of hardcore smokers attending UK general practitioners

**DOI:** 10.1186/1471-2296-7-24

**Published:** 2006-03-29

**Authors:** Hannah MacIntosh, Tim Coleman

**Affiliations:** 1 Division of Primary Care, University of Nottingham, Nottingham, UK

## Abstract

**Background:**

Smoking remains a public health problem and although unsolicited GPs' advice against smoking causes between one and three percent of smokers to stop, a significant proportion of smokers are particularly resistant to the notion of stopping smoking. These resistant smokers have been called "hardcore smokers" and although 16% of smokers in the community are hardcore, little is known about hardcore smokers presenting to primary care physicians. Consequently, this study reports the characteristics and prevalence of hardcore smokers attending UK GPs.

**Methods:**

A cross-sectional survey using data from two different research projects was conducted. Data for this analysis had been collected from surgery consultation sessions with 73 GPs in Leicestershire, England, (42 GPs from one project). Research assistants distributed pre-consultation questionnaires to 4147 adults attending GPs' surgery sessions. Questionnaires identified regular smokers, the proportion of hardcore smokers and their characteristics. Non-hardcore and hardcore smokers' ages, gender and nicotine addiction levels were compared.

**Results:**

1170 regular smokers attended surgery sessions and, 16.1% (95% CI, 14.1 to 18.4) were hardcore smokers. Hardcore smokers had higher levels of nicotine addiction than others (p = 0.000), measured by the Heaviness of Smoking Index and were more likely to be male [50.5% hardcore versus 35.3% non-hardcore, (OR = 1.88, 95% CI = 1.4 to 2.6)] but no age differences were observed between groups.

**Conclusion:**

A significant minority of the smokers who present in general practice are resistant to the notion of smoking cessation and these smokers are more heavily nicotine addicted than others. Although clinical guidelines suggest that GPs should regularly advise all smokers against smoking, it is probable that hardcore smokers do not respond positively to this and help to make up the 97%–99% of smokers who do not quit after being advised to stop smoking by GPs. General practitioners need to find approaches for raising the issue of smoking during consultations in ways that do not reinforce the negative opinions of hardcore smokers concerning smoking cessation.

## Background

Hardcore smokers are those who are especially resistant to giving up[1-3]. 16% of English smokers can be categorised as hardcore[1] as can 13.7% of US smokers[3] and 5.2% in the state of California[2]. Surveys investigating the hardcore smoking concept have used similar behavioural and motivational criteria to define it, though US studies used the additional criteria that hardcore smokers were over 26 years old and smoked at least 15 cigarettes daily. Hardcore smokers have been found to be concentrated disproportionately amongst economically disadvantaged[1] or lower income groups and are more likely to be older[1,2] and male[2,3] than other smokers. Consequently, hardcore smokers will experience substantial smoking-related health inequalities but, as they are not interested in stopping smoking and are unlikely to seek help with this, they present a public health challenge.

It has been proposed that GPs can help meet the public health challenge posed by smoking[4]. Unsolicited, brief stop smoking advice from GPs results in smoking cessation by around 1 in 50 smokers advised[5]. Consequently, GPs have been recommended to advise smokers to stop at least annually[6] and a recently-introduced contract for UK GPs rewards them for advising smokers to stop[7]. A survey of American smokers, however, found that hardcore ones were less likely to recall smoking cessation advice from a physician[3]. This could be a function of the lower consultation rates that hardcore smokers reported[3], but it is equally possible that the low motivation of these smokers deterred health professionals from discussing their smoking.

Reducing smoking prevalence within economically disadvantaged groups is necessary to reduce health inequalities and brief cessation advice from GPs is an important component of any tobacco control strategy striving for this. Some smokers, however, resent doctors' smoking cessation advice when they cannot see the relevance of this to their reason for attending the doctor[8,9] and doctors can have difficulty illustrating the relevance smoking to patients' health problems[10]. Consequently, for doctors' brief smoking cessation advice to have maximal effectiveness, GPs probably require different approaches for hardcore and motivated smokers respectively. If hardcore smokers present frequently to GPs, then the need for varied and sensitive methods for raising the issue of smoking with them becomes particularly important. Consequently, we investigated the prevalence and characteristics of hardcore smokers attending GPs' routine consultations to provide baseline information about the motivation of patients encountered by GPs in primary care.

## Methods

We used data from two previous studies[11,12]. which involved collecting information from all patients attending a selection of 73 different Leicestershire GPs' surgery consultation sessions (i.e. clinics of routine consultations). Ethical approval for the data collection in both studies was obtained from Leicestershire Ethics Committee. Studies' participants were given written information about research projects by researchers and, after reading this, their informed consent to complete questionnaires was obtained. Full details of both studies are given elsewhere[11,12] relevant summary information is presented here. No GPs participated in both studies.

### Research details

#### Study 1

This was conducted throughout Leicestershire, England in 1995/6. Participating GPs were selected from those who responded to a survey on attitudes towards brief smoking cessation advice that was sent to all Leicestershire GPs[13]. The selection method aimed to obtain doctors with varied attitudes towards discussing smoking with patients[13]. Of 123 GPs asked to participate, 53 (43.0%) agreed[14] and over the course of data collection (which took one year), 42 contributed as outlined below. A detailed comparison of participants and non-participants is given elsewhere[14]. Participating GPs had one surgery session video recorded (i.e. a clinic of routine consultations) and all data collection took place during this one session. Doctors and patients were told that the research project was interested in doctor: patient communication on preventive medicine issues. All (i.e. consecutive) patients attending data collection surgery sessions completed questionnaires (details below) upon entering GPs' surgery premises.

#### Study 2

This was conducted within an economically disadvantaged area of Leicester, England in 1998/9. All GPs in the Leicester City West area (now the area of Leicester City West Primary Care Trust) were asked to participate in an evaluation of an health promotion payment during which they could claim a fee for documenting periods of abstinence from smoking by patients and 31 (out of 62) participated[11]. The aim of this study was to determine whether or not this payment affected GPs' rates of giving brief advice against smoking (which it did not)[11]. The data collection for each GP took place at 5 or 6 randomly-selected surgery sessions during a six month period and this involved distributing pre-consultation questionnaires which were almost identical to those in Study 1 to all consecutive patients.

### Questionnaires

Questionnaires were distributed to patients by research assistants. These asked for socio-economic details, identified regular smokers (defined as smoking on at least most days) and subsequently asked regular smokers about their attitudes to smoking, smoking behaviour and levels of nicotine addiction[15]. The latter quality was measured using the Heaviness of Smoking Index, a biochemically-validated two-item measure of smoking behaviour that produces a score in the range of 0–6 with 6 representing greater nicotine addiction[15]. Questionnaires differed slightly between studies but the items used to define hardcore smokers were identical on each (see later).

**Figure 1:**
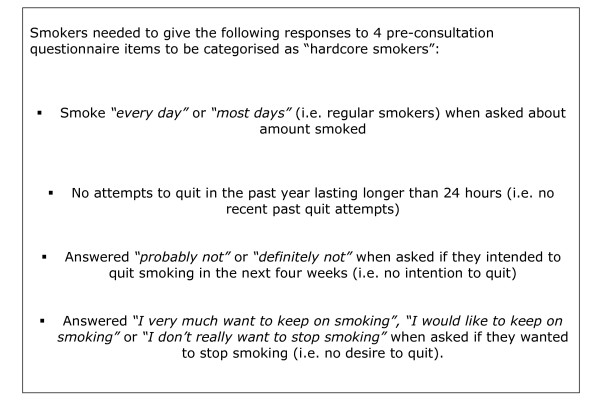
Box 1 Criteria used to define hardcore smokers.

In both studies data from pre-consultation questionnaire responses were used to determine the proportion of hardcore smokers amongst all smokers attending surgery sessions. Box 1 (Fig 1) gives details of the pre-consultation questionnaire items used to define hardcore smoking. Although there is no generally accepted definition for this, we followed the criteria used by Jarvis[1]. Jarvis used only motivational and behavioural criteria to define hardcore smoking and demonstrated a strong association between heavier nicotine addition and hardcore smoking defined in this way[1]. The one difference between our definition and Jarvis's was that, in addition to the criteria in Box 1 (Fig 1), Jarvis required hardcore smokers to have "*not gone for as much as a day without smoking in the previous five years*".

The proportions of hardcore smokers in both samples and then in the combined dataset are reported and subsequently, data provided by regular smokers within each study was analysed together. We compared the age, gender and levels of nicotine addition between hardcore and non-hardcore smokers who attended data collection surgeries in the merged dataset. Chi-square and Mann Whitney tests were used as appropriate for hypothesis testing of data.

## Results

### Sources of data: Study 1

In this study, 622 adults attended 42 general practitioners' surgeries but 4 were not included in the study as they could not read English. Of the remaining 618 surgery attenders, 99.0% (612) completed pre-consultation questionnaires and 23.5% (144) were regular smokers and were included in the merged dataset.

### Sources of data: Study 2

In the later study, 3525 patients attended data collection surgeries and 83.8% (2955) completed the pre-consultation questionnaire. 16 patients were excluded because they were unable to complete questionnaires, 97 because they refused to do so and 457 patients were missed by researchers and were not given questionnaires. Of the 2955 surgery attenders who completed pre-consultation questionnaires, 34.7% (1026) were regular smokers whose data contributed to the merged dataset. The higher prevalence of smoking in study 2 reflects the more urban, disadvantaged population.

### Merged dataset characteristics

Further analyses were conducted on the 32.8% (1170) of those from both studies who completed pre-consultation questionnaires and were regular smokers and 726/1166 (62.1%) of these were female [data missing in 4 cases]. Regular smokers had a mean (SD, IQR) age of 42.8 (15.8, 27.0 to 49.0) years and 16.1% (186/1153) [95% CI, (14.1 to 18.4)] were categorised as hardcore smokers (data missing in 17 cases). 7.9% (11/139) of regular smokers from study 1 were categorised as hardcore [data missing in 5 cases] as were 17.3% (175/1014) from study 2 [data missing in 12 cases]. The higher prevalence of hardcore smoking in study 2 probably arose because this study was conducted in a more deprived area than study 1 and there is a known association between economic deprivation and hardcore smoking[1,2].

### Comparison of hardcore and non-hardcore smokers in merged dataset

Hardcore smokers were significantly more likely to be male with 50.5% (94/186) of hardcore smokers being male versus 35.3% (341/967) of non-hardcore [OR = 1.88, 95% CI = 1.4 to 2.6]. Hardcore smokers also demonstrated higher levels of nicotine addiction as measured by the Heaviness of Smoking Index (HIS). Hardcore smokers' HIS scores were significantly higher than non-hardcore smokers' ones [Mean/median (SD/IQR) hardcore smokers' scores = 2.8/3 (1.6/2-4) versus non-hardcore scores = 2.3/3 (1.6/1-3), data missing for 4 hardcore and 23 non-hardcore smokers, Mann-Whitney U = 70297, p= 0.000]. There was no significant difference, however, between the ages of hardcore and non-hardcore smokers [mean age (SD, IQR) of hardcore smokers = 39.7 (16.4, 27.0 to 51.0) years versus 39.0 (15.6, 36.0 to 48.0) years in non-hardcore (Mann-Whitney U = 87591, p = 0.8, data missing for 1 hardcore and 7 non-hardcore smokers).

## Discussion

The principal new findings of this study are that a substantial minority of smokers attending routine general practice consultations can be categorised as "hardcore" and these non-motivated smokers are more heavily nicotine-addicted and more likely to be male than others. In this sample, 16.1% of smokers presenting to GPs' routine consultations were "hardcore" which is similar to the prevalence obtained in Jarvis' community based sample[1]. However, the research sample was not necessarily representative of UK general practice settings and, as most data collection was conducted in an area of economic disadvantage, the actual proportion of hardcore smokers presenting to GPs across the UK may be different. Nevertheless, our findings are likely to be generalisable to inner city UK settings and suggest that hardcore smoking is a prevalent phenomenon there.  Consequently, it would be feasible to deliver smoking cessation interventions to large numbers of hardcore smokers via routine general practitioner consultations in urban deprived settings.

The pre-consultation questionnaires from which we obtained our prevalence estimate had high response rates, so it is likely that our estimate of prevalence within the study sample is valid. As mentioned above, this figure is likely to differ from that which would have been obtained using a representative sample of UK general practitioners. As, the phenomenon of hardcore smoking is associated with economic deprivation[1] and most of our data came from a deprived sector of Leicester, this will probably inflate our estimate. To the authors' knowledge, there are few available data sets from which the prevalence of hardcore smokers presenting to family physicians could be calculated, however. Consequently, whilst these data have some limitations, findings should be viewed in the context of the paucity of available data on this topic. Unlike previous studies that employed community based samples we found no association between hardcore smoking and increasing age[1,2]. The mean age of hardcore smokers presenting to GPs in our sample was approximately 8 years lower than in Jarvis's survey[1]. This is probably to be expected because smokers who are able to attend GPs are generally ambulatory and younger than others. Alternatively, this difference may be explained because our criteria for defining hardcore smokers did not require smokers to have smoked for a defined time period (Jarvis stipulated smoking for the last five years).

The best approach for GPs who wish to raise and discuss the issue of smoking with hardcore smokers remains unclear. We know that when unselected (i.e. hardcore smoker and non-hardcore) smokers are given brief, simple advice against smoking, between 2 and 3% of all who are advised will stop as a consequence of this[5]. The challenge for clinicians is to introduce the topic of smoking into consultations in a way which is appropriate for individual smokers. GPs need to use a different approach with hardcore smokers than they use with those who are motivated to quit. One could argue that clinicians should not mention smoking at all with those smokers whom they perceive are not motivated to stop, but we argue that it is more appropriate to raise the issue of smoking with them in a manner that could potentially encourage them to start questioning their smoking habits. We have shown that hardcore smokers formed a significant proportion of smokers within one large sample presenting to general practitioners and that these hardcore smokers were more heavily addicted to nicotine than others. Previous work has demonstrated that these smokers are also more likely to be economically disadvantaged (and hence suffer from health inequalities) than others[1]. To ignore smoking by hardcore smokers would serve to perpetuate these inequalities and is not defensible, so there is a need for brief smoking cessation interventions which are tailored to meet their needs, are feasible to apply in primary care consultations and are acceptable for general practitioners to use. Such interventions need to engage hardcore smokers and to do this they probably should involve personalised smoking cessation messages that are appropriate for smokers who have no intention of quitting[9].

## Conclusion

Approximately 16% of smokers presenting to GPs in this research sample were categorised as hardcore smokers. These smokers are very resistant to the notion of stopping smoking, but are more highly addicted to nicotine than other smokers. GPs need to be aware of this substantial minority of smokers who present in consultations with them and are very negative about stopping smoking. When raising the issue of smoking with these patients, GPs also need to be sensitive to the low level of motivation for smoking cessation that they possess.

## Competing interests

The author(s) declare that they have no competing interests.

## Authors' contributions

TC had the idea for the study and HM worked as a medical student under his supervision. Both authors modified the study protocol, contributed to the analysis of data and wrote the paper.

## Pre-publication history

The pre-publication history for this paper can be accessed here:



## References

[B1] Jarvis MJ, Wardle J, Waller J, Owen L (2003). Prevalence of hardcore smoking in England, and associated attitudes and beliefs: cross sectional study. BMJ.

[B2] Emery S, Gilpin EA, Ake C, Farkas AJ, Pierce JP (2000). Characterizing and identifying "hard-core" smokers: implications for further reducing smoking prevalence. American Journal of Public Health.

[B3] Augustson E, Marcus S (2004). Use of the current population survey to characterize subpopulations of continued smokers: a national perspective on the "hardcore" smoker phenomenon. Nicotine & Tobacco Research.

[B4] Charlton BG, Calvert N, White M, Rye GP, Conrad W, van Zwanenberg T (1994). Health promotion priorities for general practice: constructing and using "indicative prevalences. BMJ.

[B5] Ashenden R, Silagy C, Weller D (1997). A systematic review of the effectiveness of promoting lifestyle change in general practice. Family Practice.

[B6] West R, McNeill A, Raw M (2000). Smoking cessation guidelines for health professionals: an update. Thorax.

[B7] Department of Health (2004). Investing in general practice: the new general medical services contract.

[B8] Stott NC, Pill RM (1990). 'Advise yes, dictate no'. Patients' views on health promotion in the consultation. Family Practice.

[B9] Butler CC, Pill R, Stott NC (1998). Qualitative study of patients' perceptions of doctors' advice to quit smoking: implications for opportunistic health promotion. BMJ.

[B10] Pilnick A, Coleman T (2003). "I'll give up smoking when you get me better": patients' resistance to attempts to problematise smoking in general practice (GP) consultations. Social Science & Medicine.

[B11] Coleman T, Wynn AT, Barrett S, Wilson A, Adams S (2001). Intervention study to evaluate pilot health promotion payment aimed at increasing general practitioners' antismoking advice to smokers. BMJ.

[B12] Coleman T, Manku-Scott T (1998). Comparison of video-recorded consultations with those in which patients' consent is withheld. Br J Gen Pract.

[B13] Coleman T, Williams M, Wilson A (1996). Sampling for qualitative research using quantitative methods. 1. Measuring GPs' attitudes towards discussing smoking with patients. Family Practice.

[B14] Coleman T (1996). Sampling for qualitative research using quantitative methods. 2. Characteristics of GPs who agree to video-taping of consultations. Family Practice.

[B15] Heatherton TF, Kozlowski LT, Frecker RC, Rickert W, Robinson J (1989). Measuring the heaviness of smoking: using self-reported time to the first cigarette of the day and number of cigarettes smoked per day. British Journal of Addiction.

